# New Appliances and Things Medical

**Published:** 1909-10-16

**Authors:** 


### NEW APPLIANCES AND THINGS MEDICAL.

[We shall be glad to receive at our Office, 28 & 29 Southampton Street, Strand, London, W.C., from the manufacturers, specimens of all new preparations and appliances.]

### "CONOIDS."

The Conoid Company, of 174 Corporation Street, Birmingham, has forwarded samples of their new "Conoids," which consists in plugs of cotton-wool covered with silver paper. The cotton-wool is impregnated with menthol, eucalyptol, and similar aromatic and volatile compounds, which have a decided bactericidal action. The silver foil is removed, the plug of wadding is placed in the nostril, and the scent inhaled. These "Conoids" may undoubtedly prove of great benefit in preventing colds and influenza, and may even be used with success in the incipient stages of an influenzal cold. As germicides the volatile oils take a good place in the large list of substances injurious to bacteria, and in the "Conoids" we have examined, the vapour is fairly strong and possesses a decided bactericidal action.

### STAUFFER'S "LACTO-BACTERIA" AND STAUFFER'S YEAST TABLETS

(Jean Stauffer and Co., Enghien, Belgium).

We have received and tested samples of the above preparations, which have been sent to us by the manufacturers.

The sample of *Lacto-Bacteria* was tested and found to be quite active, curdling milk in about 15 hours; and the microscopical examination of the culture showed the presence of micro-organisms similar in character to those found in the ordinary samples of "sour milks" used for therapeutical purposes. This ferment thus appears to possess the necessary properties of a substance intended for curdling milk, and as such may be recommended to the notice of the medical profession. It is prepared and sold in the convenient form of tablets, each containing 30 centigrammes. They are almost tasteless. The usual adult dose is from three to four tablets daily. Samples will be sent to medical practitioners free of charge on application to the London agent, Mr. Henry A. Riles, 28 Queen's Road, Lower Edmonton, London.

The *Yeast Tablets* supplied to us through the same source proved on laboratory investigation to contain, as alleged by the manufacturers, abundant living yeast cells. This preparation of dried yeast therefore deserves clinical trial in those cases of furunculosis and other skin affections, and perhaps also of diabetes, in which administration of the saccharomyces ferment seems indicated. Stauffer's Dry Yeast is sold in boxes of 100 compressed tablets, each tablet containing 0.25 gramme of dried yeast, which is equivalent to about 4.5 grains of fluid yeast. The usual dose is four tablets thrice a day one hour before meals. A powdered form is also sold for external and internal use. Free samples of these tablets also may be obtained on application by physicians to the British agent at the above address. Clinical reports of the results of administration of both products by Continental practitioners are also available.

### AN IDEAL ICE CUP.

The Hospitals and General Contracts Company, Limited, of 33 and 35 Mortimer Street, W., manufactures an ideal ice cup which appears to merit its name, if ever an ice cup did. The cup, which is made in various sizes, is of unbreakable metal, neatly enamelled in white, light, practically indestructible, and is easily sterilised. It possesses the indispensable perforated cover, also of enamelled tin, which can be removed and thoroughly cleansed by boiling if necessary. The price of the cup, which we consider superior by far to the usual china ice cup so frequently seen in hospitals, is 1s. 6d.

### MALVERN WATERS, ALPHA BRAND

(W. and J. Burrow, The Springs, Malvern).

We have received a sample of Malvern water, both in its natural form and also aerated. The natural water is bottled direct from the spring and is certainly a water of exceptional purity. It has a clean, sharp, refreshing taste, and contains an exceedingly low quantity of saline matter (under four grains) to the gallon. The salines consist of salts of magnesium, sodium, calcium and iron, the latter in very minute quantity. The water also contains a trace of iodide of potassium and is further entirely free from organic matter. It is almost inconceivable to imagine a purer natural water. The aerated variety is produced from the same spring, and is simply the same water aerated artificially. The importance to health of good table water, either to take by itself or to mix with wine, is so universally recognised that to insist further upon it here is unnecessary. The discolouration of red and white wines when mixed with many mineral waters depends upon the alkalinity of these latter. A water of the composition of Malvern water would have no such effect upon these wines, and a wine moderately diluted with it would lose none of that pleasant appearance and soft taste which we so appreciate in the good, light natural wines. Ordinary table water, if it is not hard, is very apt to be sapid and mawkish in taste. The continuous use of a hard water at table is often in many patients attended with injury to health and well-being; at the same time, a water to be palatable must have a certain grip on the palate. The use of a water such as Malvern water will give us all that we want in the way of taste, and by drinking it we shall at the same time avoid those unpleasant consequences which are apt to attend the habitual consumption of a hard water.

